# Flavonoids as Promising Natural Compounds in the Prevention and Treatment of Selected Skin Diseases

**DOI:** 10.3390/ijms24076324

**Published:** 2023-03-28

**Authors:** Beáta Čižmárová, Beáta Hubková, Vladimíra Tomečková, Anna Birková

**Affiliations:** Department of Medical and Clinical Biochemistry, Faculty of Medicine, Pavol Jozef Šafárik University in Košice, Trieda SNP 1, 040 01 Košice, Slovakia

**Keywords:** phytochemicals, flavonoids, skin, chronic diseases

## Abstract

Phytochemicals represent a large and diverse group of naturally occurring compounds, bioactive nutrients, or phytonutrients produced by plants, widely found in fruits, vegetables, whole grains products, legumes, beans, herbs, seeds, nuts, tea, and dark chocolate. They are classified according to their chemical structures and functional properties. Flavonoids belong to the phenolic class of phytochemicals with potential solid pharmacological effects as modulators of multiple signal transduction pathways. Their beneficial effect on the human body is associated with their antioxidant, anti-inflammatory, antimutagenic, and anticarcinogenic properties. Flavonoids are also widely used in various nutritional, pharmaceutical, medical, and cosmetic applications. In our review, we discuss the positive effect of flavonoids on chronic skin diseases such as vitiligo, psoriasis, acne, and atopic dermatitis.

## 1. Introduction

Phytochemicals (a word derived from the Greek “Phyto,” meaning plant) are naturally occurring compounds, bioactive nutrients, or phytonutrients produced by plants. These compounds are widely found in fruits, vegetables, whole grains, legumes, beans, herbs, seeds, nuts, tea, and dark chocolate. Phytochemicals are responsible for the flavor, aroma, and color of plants. Plant-based foods and beverages are associated with health benefits and reduced risks of serious chronic diseases [1,2,3]. Plants are an essential source of bioactive substances and natural antioxidants, which are very important for the pharmaceutical industry. Their importance continues to grow due to their function in preventing and treating many human diseases. Recent developments show a positive effect and improvement in the acceptance of pharmaceutical products based on medicinal and functional foods in all aspects of human life [4,5].

**Figure 1:**
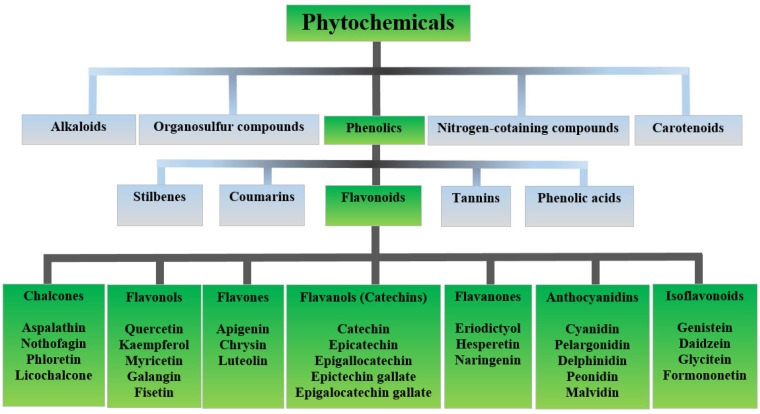
Classification of dietary phytochemicals with emphasis on the flavonoid group (Adapted from [7]).

Phytochemicals are a large and diverse group of chemicals, with tens of thousands of phytochemicals isolated and identified from plants [3,6]. They are divided into groups and subgroups according to their chemical structure and functional properties [1], namely carotenoids, phenolics, alkaloids, nitrogen-containing compounds, and organosulfur compounds (Figure 1) [7]. Of all the mentioned, phenolics belong to the most studied group of phytochemicals [2]. Phenolic compounds are reducing agents, and all of them act as powerful antioxidants, playing important roles through different mechanisms such as the modulation of the activity of antioxidant enzymes, chelation of metal ions (Fe, Cu, and others), and anti-inflammatory reactions [8,9].

Phenolic compounds are a large and varied group of secondary metabolites in fruits and vegetables. Plant and plant fruits are important sources of phytochemicals, but fruit peels also contain significant phytochemicals, including vitamins, dietary fibers, carotenoids, and phenolic compounds. There is a considerable amount of waste that is generated during the processing of fruit in the food processing industry, mainly peels, seeds, and some other fruit residues [10]. The amount and the type of polyphenols vary between the fruit peel; however, among the most abundant are flavan-3-ols, flavonols, phenolic acids, anthocyanins, and hydroquinones [11].

Suleria et al. [12] determined polyphenol content in twenty different fruit peel samples, including peels from apples, apricots, avocadoes, bananas, custard apples, dragon fruits, grapefruits, kiwifruits, limes, mangoes, melons, nectarines, oranges, papayas, pears, peaches, pineapples, passionfruit, plums, and pomegranates. Obtained results pointed out the importance of fruit peels as a potential polyphenols source. Their results showed that the highest total phenolic content was observed in mango peels, followed by citrus fruits’ (grapefruit, orange, lime) peel. The highest total flavonoid content was observed in mango peels, followed by pineapple and banana peels. The highest total tannin content was again in the mango and avocado peels. Among the peels commonly consumed with fruit, apple, apricot, pear, and peach peels had the highest flavonoid content, and nectarine peels had the lowest [12]. Berry fruit and berry fruit leaves contain many bioactive natural compounds. Berry leaves are considered a by-product of berry cultivation despite containing similar bioactive compounds to berry fruits (flavonols, anthocyanins, phenolic acids, phenolic acids esters, and procyanidins). Berry leaves are also a rich source of chlorogenic acid. Although berry leaves are used in treating several diseases (e.g., urinary tract infections, colds), tons of berry leaves are disposed of as a by-product every year [13]. Other good sources of bioactive compounds are plant seeds that contain carotenoids, tocopherols, xanthophylls, and polyphenols such as phenolic acids, flavonoids, stilbenes, and lignans. Plant seeds are also important dietary sources of minerals and nutrients such as carbohydrates, lipids, and proteins [14].

The skin is the largest organ of the human body, protecting the entire organism against external threats such as mechanical damage, radiation, allergens, chemicals, and infections. The weakening of this barrier by chronic diseases has far-reaching consequences for various metabolic processes and significantly influences the already weakened health of the affected people. The associated negative self-image often leads to decreased quality of life, loneliness, and psychological disorders. In addition, recent studies show that environmental factors are crucial in increasing the incidence of autoimmune diseases, including skin manifestations [15]. These include, for example, vitiligo, scleroderma, lupus, vasculitis, and psoriasis. According to Chang et al.’s retrospective cohort study, COVID-19 is associated with a different degree of risk for various autoimmune diseases [16].

The present review aims to summarize flavonoids’ beneficial antioxidative, antiallergic, and photo-protective effects on the skin in chronic diseases such as vitiligo, psoriasis, acne vulgaris, atopic dermatitis, and skin cancer. These phytochemicals have a perspective in designing modern drugs, treatments, and even the prevention of skin diseases. Generally, the development of synthetic analogs is progressing by leaps and is based on imitating natural sources but with improved functions. However, flavonoids are abundant in nature without apparent side effects. Furthermore, plant-derived treatments are more affordable due to their natural availability. For their effect to be demonstrated in the best possible way, an increase in their bioavailability after being accepted by the human body is necessary.

## 2. Flavonoids

Flavonoids are the most abundant and widespread secondary plant metabolites in fruits and vegetables. They represent the most isolated, the most studied, the most identified, and the most diverse class of polyphenolic compounds [17]. Flavonoids are responsible for the fragrance and color of flowers. They are responsible for many health benefits and are considered dietary supplements supporting health and disease prevention. Flavonoids are potent antioxidants with antiviral, antibacterial, anti-inflammatory, and antiallergic properties. They are also essential to many pharmaceutical nutraceuticals, medicinal, cosmetic, and other applications [17,18]. Currently, more than 10,000 flavonoids are known and divided into different subgroups [19]. 

**Figure 2:**
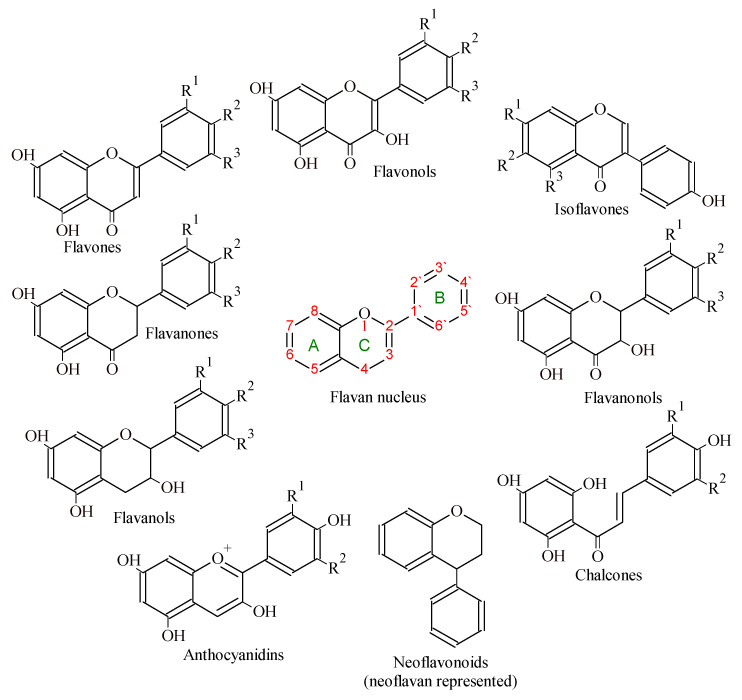
Chemical structure of the basic flavonoid skeleton and derived groups of flavonoids.

Their structure (Figure 2) is based on the flavan nucleus, consisting of two benzene rings (ring A and ring B) and the pyran ring (ring C) [17,20]. The classification depends on the number and the type of substituents on the last outermost ring, on the number and position of hydroxyl groups in the rings, and also on the presence/absence of a double bond at position four of ring C and a double bond between C2 and C3 and hydroxyl groups in ring B [17,21]. Flavonoids can be divided into subgroups depending on the carbon of the C ring to which the B ring is attached (Figure 2). In case ring B is connected to ring C in position two of the C ring, the basic flavonoid structure can be subdivided into subgroups according to the degree of unsaturation and oxidation of the C ring, namely to flavones, flavonols, flavanones, flavanonols, flavanols or catechins, anthocyanidins, and chalcones. If the bond between the B ring is moved from position two to position three of the C ring, isoflavones are formed; if to position four of the C ring, neoflavonoids are formed. Flavonoids are also in the form of aglycones, glycosides (O- or C-), and methylated derivatives [22,23,24,25]. The most important plant sources of the listed flavonoids are summarized in Table 1.

### Flavonoids in Cosmetic Application

An interest in using natural compounds in cosmetics is still increasing. Between 2015 and 2019, the global market dealing with natural cosmetics was expanding, with an annual growth of about 10–11%. Additionally, in the 21st century, there is still a growing trend to use naturally derived ingredients in cosmetic applications [61]. Cosmetics products are designed for application on the skin, hair, and body, while the main aim is cleansing, beautifying, promoting attractiveness, or improving the appearance [62]. An essential factor in evaluating the safety of botanical ingredients in cosmetics and personal care products is the phytochemical characterization of the plant source, contamination data, dangerous residues, and adulteration. Botanical ingredients that are used in the cosmetic industry could be produced by different processing methodologies, such as plant extracts, expressed juices, tinctures, waxes, vegetable oils, lipids, plant carbohydrates, essential oils, as well as purified plant components, such as vitamins, antioxidants, and other substances with recognized biological activity [63]. The new term—cosmeceutical is defined as cosmetic products containing biologically active ingredients. The name cosmeceutical comes from two words: cosmetic and pharmaceutical. Biologically active ingredients present in cosmetics include peptides, polysaccharides, phytochemicals, vitamins, antioxidants, and essential oils. The listed compounds positively affect human skin, such as UV protection, whitening, tanning, anti-wrinkling, antiaging, and many others [64]. Plant extracts are rich in secondary plant metabolites representing a large and diverse group of compounds with high structural diversity. Polyphenols have gained increasing prominence from all the components found in botanical preparations for cosmetic use due to the plethora of biological activities. Phenolic compounds (flavonoids and non-flavonoids) are associated with numerous cosmetic properties, e.g., photoprotection, antiaging, moisturizing, antioxidant, astringent, anti-irritant, and antimicrobial activity. It was confirmed that plant extracts could potentially prevent premature skin aging evoked by oxidative stress [62]. If talking about polyphenolic compounds, after application, they exhibit an anti-inflammatory effect and can inhibit the gene expression and activity of skin enzymes (e.g., hyaluronidase, matrix metalloproteinase, collagenase, serine protease elastase) [61,62,65]. The anti-inflammatory activity of flavonoids is commonly used in the field of cosmetology. It is well known that flavonoids act as antioxidants and are very effective as free radical scavengers. Flavonoids inhibit the release of arachidonic acid caused by oxidative processes of membrane lipids. Flavonoids with chelation properties inactivate 5-lipoxygenase and cyclooxygenase, which are important in transforming arachidonic acid into proinflammatory leukotrienes and prostaglandins [66]. Their antioxidant properties correlate with their activity on the skin and their soothing properties [67]. There is increasing evidence about using polyphenolic compounds in sunscreens due to their ability to absorb UV light and their photostabilizing effect [62]. Flavonoids, thanks to their antiradical properties, are capable of absorbing ultraviolet radiation in a wide range, with maximum far ultraviolet B (250–280 nm) and A (350–385 nm) [68].

## 3. Skin as a Barrier

Skin covers the entire external surface of the body and is considered the largest human organ [69]. Human skin provides immunological protection and physical integrity from the external environment [70]. In adults, the skin covers an area of about 2 m^2^, its average thickness is about 2.5 mm, and the average density is 1.1. The skin represents up to 6% of total body weight [71]. The skin’s structure provides a physical barrier at the interface with the external environment, skin is made up of distinct layers (the epidermis, dermis, and hypodermis) and cell types, and it protects the body against UV radiation, chemicals, mechanical injury, microbial infection, and pathogens. Skin also regulates temperature and the amount of water released into the environment [69,72,73]. The skin contains many low molecular-weight compounds with specific physiological roles [74].

The external superficial skin layer is the epidermis. The epidermis represents the first protection barrier against the invasion of foreign substances. It is continually keratinizing stratified epithelium that terminates at mucocutaneous junctions. It comprises the basal layer—stratum basale (which contains keratinocytes), followed by stratum spinosum, stratum granulosum, and stratum corneum (with biochemically active cells—corneocytes). Fundamental cells of the epidermis are keratinocytes. Langerhans cells, melanocytes, Merkel cells, and T-lymphocytes are other cells in this layer. The epidermis protection barrier function depends on transglutaminase-mediated cross-linking of structural proteins and lipids during terminal stages of differentiation of keratinocytes [75]. The stratum corneum barrier quality is associated with the presence of equimolar amounts of ceramides, cholesterol, and free fatty acids [76]. Human skin permeability is based on the quality and quantity of lamellar lipid domains located between the corneocytes (stratum corneum). Fatty acids produced endogenously are also found in the lipid between the corneocytes (stratum corneum) layers and in the so-called hydrolipidic film on the skin’s surface. These biomolecules are critical contributors to the structure–function relationship of the skin’s epidermis [77]. The epidermis is a renewing layer in which basal keratinocytes (from stratum basale) are transformed into corneocytes (stratum corneum) in a process that takes approximately 30 days [73].

The dermis is divided into two areas: the superficial papillary region and the deeper reticular region. Compared with the epidermis, the dermis contains a lower amount of cells but higher amounts of fibrous and the amorphous extracellular matrix [78]. The primary cell types of the dermis are fibroblasts, and multifunctional cells of the immune system such as macrophages and mast cells. Many extracellular matrix components such as collagens, proteoglycans, fibronectin, fibrin, and glycosaminoglycans are considered the protagonists of fibroblast metabolism, survival, and migration [79]. The principal component of the extracellular matrix is collagen; other components are elastin, fibrillin, fibullins, integrins, laminins, and proteoglycans [80].

The third layer—hypodermis, also called subcutaneous fascia, is below the dermis. Its main components are adipose lobules. It also contains skin appendages (hair follicles, sensory neurons, and blood vessels). Its principal function is heat insulation, mechanical cushion, and storage of readily available high-energy chemicals [69,81].

### Oxidative Stress-Association with Skin Diseases

The skin is a very important organ, representing the barrier—the first interface between the human body and its environment. It protects the human body against physical and chemical effects and injuries. Exposition of the skin to UV light, pollutants, and allergens leads to the production of free radicals in the skin and the formation of oxidation stress promoting and developing a variety of skin diseases, causing changes in skin homogeneity, sagging, wrinkling, roughness, and dryness [21,82].

Oxidative stress is widely known as an imbalance between the accumulation of free radicals/oxidants and the antioxidant system in the human body. Oxidative stress leads to DNA damage, lipid peroxidation, protein oxidation, and a series of activation/inactivation of signaling pathways, which contributes to the occurrence and development of many skin diseases, e.g., psoriasis, vitiligo, skin photodamage, skin cancer, systemic sclerosis, chloasma, atopic dermatitis, and pemphigus [83]. The free radicals include reactive oxygen species (ROS), reactive nitrogen species (RNS), reactive sulfur species, and reactive carbon species. Antioxidants in the human body neutralizing free radicals are divided into two main groups: antioxidant enzymes (superoxide dismutase, catalase, glutathione peroxidase) and non-enzymatic antioxidants (e.g., glutathione, ascorbic acid, tocopherol) [84]. ROS and RNS (superoxide anion radical (O_2_^−•^), hydroxyl radical (^•^OH), singlet oxygen (^1^O_2_), hydrogen peroxide (H_2_O_2_), nitric oxide (NO), and peroxynitrite (ONOO^−^)) are also produced during intracellular metabolic processes. Production in controlled concentrations exhibits beneficial effects on the human body. ROS and RNS contribute to some physiological processes; for example, cell signaling, gene transcription, and neurotransmission [85]. Their increased concentrations lead to oxidative stress associated with several human diseases, including dermatologic diseases, skin wrinkling, and the pathogenesis of human skin cancer. ROS participate in inflammatory and allergic skin disease pathogenesis [86]. In mammalian cells, mitochondria are the primary source of ROS, which are produced as byproducts of normal mitochondrial metabolism in the reactions of the respiratory chain [82,86]. Exogenous sources such as UV rays, pollutants, and stress also produce ROS in the skin. The composition of skin microbiota associated with healthy skin is also affected by UV radiation and pollutants [82]. The type of ROS produced by UV rays depends on UV radiation energy. Based on the interaction of UV radiation wavelengths with biological materials, three types of UV radiation are known: UVA (400–315 nm), UVB (315–280 nm), and UVC (280–100 nm) [87]. UVA generates mostly ^1^O_2_ and also O_2_^−•^. UVB generates O_2_^−•^ predominantly [85]. It was also reported that visible light (400–700 nm) could produce ROS in the skin, namely ^•^OH, ^•^OOH, and ^1^O_2_ [88]. As mentioned above, the formation of ROS in the skin is affected by various pollutants, including gases such as ozone and particulate matter originating from fuel combustion containing polycyclic aromatic hydrocarbons (PAH). Some PAH can induce strong oxidative stress under UVA exposure. Pollution can worsen some skin diseases, such as atopy or eczema. Data also report the correlation with the early occurrence of (photo)-aging markers. A combination of skin surface protection (antioxidants, long UVA sunscreens) is recommended as the preventive strategy [89]. The human skin also hosts innumerable micro-organisms, known as cutaneous or skin microbiota, with various functions, such as bi-directional interactions with the epidermal barrier and systemic immunity. Cutaneous microbiota could represent the boundary between external exposures and the epidermal barrier. Cutaneous microbiota are important for skin health and dermatological conditions and are also connected with aging. Microbiota may be affected by exposure to UV radiation, which is related to immunosuppression induced by UV [90,91]. 

## 4. Flavonoids in Selected Oxidation Stress-Related Skin Diseases

Healthy skin is essential for the quality of human life. Skin diseases influence the quality of a patient’s life and psychological well-being. Therefore, searching for and finding effective therapeutic compounds without or with minimal adverse side effects is very important. In the research area, there is increasing evidence of flavonoid benefits in managing skin disorders, including psoriasis, acne, atopic dermatitis, urticaria, ringworm, and others [21]. Natural products seem to be a perspective in preventing and treating many skin diseases due to their wide range of pharmacological activities. Our work focused on flavonoids’ great potential in treating selected skin diseases—vitiligo, psoriasis, acne, and atopic dermatitis. Flavonoids, natural plant extracts, belonging to powerful exogenous antioxidants, are widely used in cosmetics, and are always mixtures containing aglycones and lipophilic glycosides. Such a chemical structure increases their antioxidant properties and allows them to scavenge almost all free radical types. They have a high affinity for singlet oxygen and reduce tocopheryl- and tocotrienol anion radicals. Flavonoids inhibit various ROS formation factors, thus preventing skin aging [66].

### 4.1. Flavonoids in Vitiligo Treatment

Vitiligo is an autoimmune skin disorder in which the destruction of melanocytes leads to the loss of pigmentation on the skin surface and mucosa, subsequently leading to the gradual expansion of the discolored skin area [92]. There is strong evidence that oxidative stress is an essential factor in the incidence and progression of vitiligo because it is responsible for melanocyte molecular dysfunction, melanocyte-specific antigen exposure, and melanocyte cell death [93]. The etiology of this disease is still unclear; much evidence supports the significant impact of oxidative stress on the development of vitiligo. Many exogenous and endogenous stimuli worsen the stress of melanocytes, leading to the overproduction of H_2_O_2_, thereby triggering oxidative stress to impair Nrf2 signal activation in vitiligo melanocytes. The membrane peroxidation and accumulation of ROS peroxidation is already detected in melanocytes and keratinocytes within uninvaded vitiligo parts. In addition, cytokines IL-1, IL-6, and TNF significantly increase the expression of IL-8 in melanocytes inducing oxidative stress and keratinocytes and melanocytes apoptosis [83]. This disorder has worldwide prevalence, regardless of age and gender. There are different methods; however, there is no specific treatment for vitiligo [94]. Natural compounds could be promising in vitiligo treatment as natural products promote melanin production and prevent it from destruction. The most important activity of natural compounds is their possibility to scavenge free radicals, activate pathways related to melanogenesis, increase tyrosinase gene expression, decrease chemokine and inflammatory cytokine expression, and prevent CD8+ T cell migration [93].

***Quercetin*** (3,3′,4′,5,7-pentahydroxyflavone) belongs to flavonols, a member of flavonoids. Research studies show the positive effect of quercetin in vivo and in vitro treatment of pigmentary disorders. Quercetin has the potential to protect melanocytes and keratinocytes from oxidative stress. Additionally, the topical application of quercetin may prevent damage to the cells caused by ultraviolet radiation [95]. H_2_O_2_, as one of the ROS, can induce endoplasmic reticulum expansion and prevent the production of functional tyrosinase from the endoplasmic reticulum of melanocytes, leading to the pathogenesis of vitiligo. Quercetin can reduce the series of oxidative reaction processes mediated by H_2_O_2_ and ultimately reduce the incidence of vitiligo [42]. An effect of quercetin on melanogenesis was investigated at different doses (1, 5, 10, 20 μm) and for different times (1, 3, 5, 7 days) in cultured cells, namely HMVII melanoma cells and human epidermal melanocytes (NHEM). It was demonstrated that quercetin stimulated melanogenesis by increasing tyrosinase activity and decreasing other factors, such as melanogenic inhibitors. Dose and time-dependent increase in melanin content was observed [96]. The melanogenic effects of 14 selected flavonoids were evaluated to investigate the correlation between their chemical structures and melanogenic impacts. It was deduced that the hydroxyl group bound to the phenyl ring is important in stimulating melanogenesis. Tested flavonols ***quercetin, kaempferol, rhamnetin, and fisetin***, the flavones represented by ***apigenin, luteolin***, and ***chrysin***, and isoflavones represented by ***genistein*** showed melanogenesis-promoting actions, but on the other hand, ***rutin, robinetin, myricetin, epigallocatechin gallate***, and ***naringin*** did not. Eight flavonoids (quercetin, kaempferol, rhamnetin, fisetin, apigenin, luteolin, chrysin, and genistein) enhanced tyrosinase activity in HMVII cells by inducing tyrosinase protein expression [29]. There is much evidence about the positive effect of ***kaempferide***, a monomethoxyflavonol which is the 4′-O-methyl derivative of kaempferol. It was shown that kaempferide significantly increased the expression of melanin-biosynthetic genes (MC1R, MITF, TYR, TYRP1, and DCT) and the tyrosinase activity in the B16F10 melanoma cell line. Kaempferide has no apparent cytotoxic effect on the B16F10 melanoma cell line at the concentration of 16–32 μm for 24 h [97].

### 4.2. Flavonoids in Psoriasis Treatment

Psoriasis is a chronic, immune-mediated, disfiguring, painful, and disabling skin disease. The etiology of psoriasis is unclear, but the leading causes are an imbalance of genetic, immunological, and environmental factors and the involvement of oxidative stress [98,99,100]. There is also evidence about the development of psoriasis by external and internal triggers. Many research studies demonstrated that psoriasis triggers, including mild trauma, sunburn, infections, systemic drugs, stress, cigarette smoking, air pollution, physical damage, and biological agents (e.g., viruses, bacteria), could contribute to damage of keratinocytes [100,101]. It was also demonstrated that ROS and NOS are involved in the pathogenesis of psoriasis; therefore, redox imbalance and elevated levels of inducible NOS are responsible for oxidative stress formation [102]. Psoriasis can occur at any age, but mostly at 50–69. With a global prevalence of 2–3%, psoriasis influences over 125 million people [100,103]. The most common form of psoriasis, psoriasis vulgaris, is characterized by almost symmetrical red and scaly plaques and papules covered with white or silver scales, especially on the surfaces of the extensors, the scalp, and the lumbosacral region [99,103]. Hyperproliferation and aberrant differentiation of keratinocytes and infiltration by inflammatory cells can be observed in histological findings of psoriasis [101]. General psoriasis treatments include phototherapy, photochemotherapy, and immunosuppressive drugs (methotrexate and cyclosporine). In the treatment of pustular psoriasis, retinoids are used. Psoriasis is treated with local preparations that contain salicylic acid, urea, tar, glucocorticosteroids, and vitamin D3 derivatives [21]. There is also evidence of the positive effect of flavonoids in psoriasis treatment. Current medications, a healthy lifestyle, and the integration of a diet rich in antioxidants seem promising in reducing oxidative stress damage caused by psoriasis, especially at the skin level [98]. The hyperproliferation of keratinocytes and chronic inflammation in psoriasis is associated with increased expression of the tumor necrosis factor (TNF) and vascular endothelial growth factor (VEGF). 

***Luteolin’s*** (3′,4′,5,7-tetrahydroxyflavone) effect on psoriasis lesions was tested using normal human epidermal keratinocytes and using human keratinocytes (HaCaT) cells. The experiment shows that pretreatment by luteolin (1–100 μM) significantly inhibits mRNA expression and the release of IL-6, IL-8, and VEGF in a concentration-dependent manner. Luteolin also decreases TNF-induced phosphorylation, nuclear translocation, and DNA binding of the nuclear factor-kappa B (NF-κB) [30]. The effect of luteolin was also described in another study, where its effect was tested against imiquimod (IMQ)-induced psoriasis-like lesions on BALB/c mice. It was observed that luteolin improved psoriasis-like skin lesions via suppressed infiltration by immune cells and downregulation of IL-6, IL-1β, TNF-α, IL-17A, and IL-23 expression. The anti-inflammatory activity was accomplished by inhibiting NF-κB expression and activation. The results also showed that luteolin application suppressed nitric oxide, iNOS, and COX-2 [31]. 

***Delphinidin*** (3,3′,4′,5,5′,7-hexahydroxyflavylium), representative of anthocyanidins, also showed a positive effect in psoriasis treatment. Topical application of delphinidin (0.5 mg/cm^2^ and 1 mg/cm^2^ skin areas, respectively) was performed on five-week-old female homozygous flaky skin mice, five times a week, up to 14 weeks of age. The application of delphinidin reduced the level of pathological markers of psoriatic lesions and inhibited inflammation. The induction of caspase-14, reduction of infiltrating macrophages, and decrease of keratin 14 causing hyperproliferation were observed [56]. The effect of delphinidin (0–20 μM; 2–5 days) was also evaluated by using a three-dimensional reconstructed human psoriatic skin equivalent model. Results showed that delphinidin induced cornification without affecting apoptosis and the mRNA and protein expression of differentiation markers (caspase-14, filaggrin, loricrin, involucrin). Delphinidin application decreased the expression of proliferation and inflammation markers [57]. 

***Baicalein*** (5,6,7-trihydroxyflavone), a flavone originally isolated from the roots of *Scutellaria baicalensis* and *Scutellaria lateriflora*, was topically applied for four days to the psoriatic lesions of BALB/c mice, after a 5-day topical imiquimod application. After, baicalein application was observed to significantly improve in the erythema, scaling, and thickness of the epidermal layer. The levels of IL-17A, IL-22, IL-23, and TNF in the skin significantly decreased [32]. Treatment of male Balb/c mice with *S. baicalensis* extract significantly reduced the thickness of the epidermis and attenuated psoriatic lesions, and inhibited the activation and infiltration of macrophages by alleviating inflammatory factors such as NF-κB and COX-2 [33]. Similar results were achieved by topical application of ***astilbin*** isolated from the rhizome of Smilax sp. in the study of Xu et al. [54].

***Quercetin*** also shows a significant antipsoriatic effect. Quercetin in doses 30, 60, and 120 mg/kg for seven days in IMQ-induced mice significantly decreased TNF-α, IL-6, and IL-17 levels, increased GSH, CAT, and SOD activities, and decreased the MDA (malonedialdehyde) accumulation in skin tissue induced by IMQ in mice [43].

### 4.3. Flavonoids in Acne Vulgaris Treatment

Acne vulgaris is one of the most common chronic inflammatory dermatoses affecting the pilosebaceous unit. Acne occurs in both males and females, but it dominantly affects adolescents and young adults in more than 85% [34,104]. There is evidence that acne can persist into adulthood. The prevalence of acne is 50.9% in women ages 20–29 years versus 26.3% in women ages 40–49. Acne leads to the morbidity associated with residual scarring and psychological disorders such as depression and anxiety, negatively impacting quality of life [105]. The main clinical manifestations of acne lesions are non-inflammatory (open and closed comedones) or inflammatory (papules and pustules), which occur primarily on the face, neck, trunk, and back. The pathogenesis of acne involves hyperplasia and hypersecretion of sebaceous glands, altered keratinization, inflammation, and bacterial colonization of hair follicles by *Propionibacterium acnes* [106,107]. Treatment of acne includes topical application of benzoyl peroxide, retinoids, antibiotics, and its combination. Although there is no ideal procedure for treating acne, a suitable regimen for reducing lesions can be found for most patients. Furthermore, more high-quality evidence still needs to be found on the comparative effectiveness of standard topical and systemic acne therapies [45]. 

A positive antibacterial effect of flavonoids against P. acnes was reported. It was found that licorice flavonoids—***licochalcone A, licochalcone C, licoflavone A, neobavaisoflavone, liguiritigenin, isoliquiritigenin***—play an anti-acne role by inhibiting PI3K-Akt signaling pathways and mitochondrial activity [34].

***Quercetin*** significantly suppressed proinflammatory cytokines production in *P. acnes* stimulated HaCaT, THP-1, and RAW 2647 cell lines. Quercetin reduced TLR-2 expression, MMP-9 mRNA levels, and phosphorylation of MAPK. In *an* in vivo study, *P. acnes* was intradermally injected into mice’s ears, and quercetin treatment markedly reduced ear thickness and swelling [45]. Quercetin-loaded nanovesicles called asposomes (ascorbyl palmitate vesicles) were developed and used in acne treatment. Their antibacterial activity was evaluated on *P. acnes* using 3T3 CCL92 cell lines. Prepared asposomes had an appropriate size (125–184 nm) and showed a more substantial antibacterial impact against *P. acnes* than quercetin alone. Quercetin asposomes used in 20 patients suffering from acne reduced inflammatory lesions, comedones, and total lesions [46].

The effects of ***kaempferol*** and ***quercetin*** and their combinations with antibiotics erythromycin or clindamycin were evaluated against antibiotic-resistant *P. acnes*. Antibacterial properties of quercetin and kaempferol were demonstrated with minimal inhibitory concentrations for both compounds < or =32 μg/mL and < or =64 μg/mL. It was determined that combining clindamycin with kaempferol or quercetin showed a better synergic effect than combining erythromycin with kaempferol or quercetin [44]. The promising positive effect of ***kaempferol*** and (+)- **catechin** in acne treatment showing the inhibitory effect on *P. acnes* GehA (glycerol-ester hydrolase A) was demonstrated in another study [108]. 

Green tea extract (57% ***epigallocatechin gallate***—EGCG and 16% ***epicatechin gallate***—ECG, with minor levels of other catechins) lowered the acne lesions on the nose, perioral area, and chin. Green tea extract was administrated in capsules (500 mg extract per capsule) for four weeks in the study group (40 subjects) that received 1500 mg of green tea extract daily compared to the control group (40 subjects) receiving cellulose as the placebo [109]. EGCG in human SEB-1 cells reduced sebum production by modulating the AMPK/SREBP-1 signaling pathway, reduced inflammation by suppressing NF-κB and AP-1 pathways, induced cytotoxicity of SEB-1 sebocytes via apoptosis, and decreased the viability of P. acne. A positive effect of EGCG was observed in an 8 week randomized clinical trial, where significantly improved acne was observed [110].

***Nobiletin***, a citrus polymethoxyflavonoid, improves acne treatment through mechanisms involving inhibition of diacylglycerol acyltransferase-dependent triacylglycerol synthesis, sebocyte proliferation, and progressive sebum secretion, which is independent of apoptosis but dependent on protein kinase A-activated phosphatidylserine externalization [35].

### 4.4. Flavonoids in Atopic Dermatitis Treatment

Atopic dermatitis, or atopic eczema, is a chronic relapsing inflammatory skin disease. It is a complex disease with a broad spectrum of clinical presentations and combinations of symptoms such as permanent itching, red or brownish patches of skin, dry skin, and cracked or scaly skin. Eczema usually appears as small bumps on the cheeks, rashes on the knees or elbows (often in the creases of the joints), on the back of the hands, or the top of the head. Atopic dermatitis is associated with allergy, asthma, and allergic rhinitis [111,112]. The incidence and prevalence of atopic dermatitis are still increasing; there are significant differences between children and adults, usually showing that children have a higher prevalence than adults [113]. Atopic dermatitis affects up to 20% of children and up to 3% of adults [112]. Accumulated evidence about the pathogenesis of atopic dermatitis agrees that it is a complex and multifactorial process associated with genetic defects and immunological disorders, environmental triggers, and defects of the epidermal barrier function that contribute to the penetration of allergens and infectious agents, which leads to further skin inflammation and allergic sensitization [112,113,114,115]. Important to mention is that the normal function of the skin barrier (e.g., regulation of transepidermal water loss, and protection against the action of external physicochemical agents and microorganisms) is dependent on epidermal barrier structures such as filaggrin, loricrin, keratin fibers, lipids, corneodesmosin, and kallikreins [116]. Two main hypotheses explain the causes of atopic dermatitis: the inside-out and outside-in hypotheses. The first, inside-out hypothesis, suggests that inflammation precedes barrier impairment. The disruption of the skin barrier subsequently leads to an increased penetration of allergens and microbes, their introduction, and their presentation and worsening inflammatory reaction. The second theory, outside-in, explains that atopic dermatitis is preceded by a disrupted skin barrier, which is necessary for immune dysregulation to occur. For example, the downregulation of filaggrin, required for proper skin barrier function, could make the skin more susceptible to immune dysregulation, ultimately leading to atopic dermatitis [117]. Atopic dermatitis has two different phases: acute and chronic phase. In the acute phase, skin lesions are infiltrated with CD4+T cells, which mainly secrete the Th2 (T helper) cytokines IL-4, IL-5, and IL-13. The acute phase is linked to Th2, Th22, and Th17 cell activation. During the chronic phase, Th1 cells secrete interferon IFN-γ. The chronic phase shows Th1-type inflammation and delayed-type hypersensitivity due to an IFN-γ response that induces tissue remodeling, skin thickening, and elevated collagen deposition [50,116,118,119]. There are many pharmacological therapies used in the treatment of atopic dermatitis, including corticosteroids, topical calcineurin inhibitors (tacrolimus, pimecrolimus), antihistamines, and systemic immunosuppressants (cyclosporine, methotrexate, azathioprine) [115,120,121]. However, these medicaments have some limitations and also adverse side effects. Therefore, there is increasing demand in the use of natural plant products. ***Liquiritigenin***, a dihydroflavonoid component, was used in in vitro and in vivo experiments. In vitro experiments showed that pretreatment of Jurkat T cells with liquiritigenin decreased the production of IL-2 and expression of CD69 on the surface of stimulated cells with PMA/A23187 or anti-CD3/CD28 antibodies via the NF-κB and MAPK pathways. Liquiritigenin also reduced the expression of surface molecules (CD40L and CD25) involved in the late phase of T-cell activation. In vivo, experimental data showed that oral administration of liquiritigenin reduced the redness and swelling of draining lymph nodes (dLNs) in mice and had a systemic influence on the expression levels of effector cytokines exacerbating atopic dermatitis, including IL-4, IL-5, IL-13, IL-31, TNF-α, and IL-17 [51]. ***Naringenin*** effects on skin inflammation, proinflammatory cytokines, and M1 to M2 macrophage polarization shifts were investigated in atopic dermatitis NC/Nga mouse mode. The treatment effectively suppressed the expression of the M1 marker CD68 and modulated HMGB1, RAGE, ERK1/2, and NF-κB p65 expression in the skin of atopic dermatitis mice. Naringenin treatment significantly restored M2 phenotypes, including IL-10 and CD36 levels. Results demonstrated that naringenin activates the anti-inflammatory gene [52]. The therapeutic effect of naringenin on 2,4-dinitrofluorobenzene-induced atopic dermatitis in NC/Nga mice mode was studied. It was demonstrated by Kim et al. (2013) that naringenin decreased the atopic dermatitis skin lesions’ growth by inhibiting the formation of interferon-gamma (IFN-γ) by activated CD4+ T-cells and also through the reduction of the infiltration of skin lesions through CD8+ T-cells, CD4+ T-cells, mast cells, and eosinophils. The authors also observed an improvement in ear swelling in the naringenin-treated group of mice following a histological analysis of the epidermis thickness [50]. 

***Quercetin*** effect on atopic dermatitis (AD)-like skin lesions was examined in an MC903-induced AD mouse model. The left ear of mice was applied with MC903, followed by Quercetin administration daily on the ear for eight days. Quercetin significantly suppressed atopic dermatitis acuteness and model ear epidermis thickness. It can also inhibit the infiltration of mast cells in the skin lesions and reduce the expression levels of CCL17, CCL22, IL-4, IL-6, IFN-γ, and TNF-α. Results of the study showed that the protective function of quercetin in an atopic dermatitis mouse model was mainly exhibited by regulating mast cells, keratinocytes, and Th1/Th2 cells [47].

***Chrysin*** (5,7-dihydroxyflavone) was used in the atopic dermatitis BALB/c mice model, where repeated alternative treatment of 2,4-dinitrochlorobenzene/Dermatophagoides 12hosph extract caused AD-like skin lesions. Oral administration of chrysin decreased ear thickness, also confirmed by a histopathological analysis. The study’s results demonstrated that chrysin decreased infiltration of mast cells, reduced serum histamine levels, and suppressed atopic dermatitis by inhibiting the inflammatory responses of Th1, Th2, and Th17 cells in mouse lymph nodes and ears. Experiments performed on HaCaT cells showed that chrysin inhibited TNF-α/IFN-γ-stimulated IL-33 expression. It was also demonstrated that chrysin significantly inhibited the production of cytokines, Th2 chemokines, CCL17, and CCL22 by the down-regulation of p38 MAPK, NF-κB, and STAT1 in tumor necrosis factor (TNF)-α/interferon (IFN)-γ-stimulated human keratinocytes [122]. Downregulation of the inflammation-related gene expression and upregulation of the skin-related gene expression on TNF-α and IFN-γ induced HaCaT cells was achieved using tamanu oil, rich in neoflavonoids ***calophyllolide, dalbergichromene, dalbergin, nivetin, and coutareagenin*** in a study of Tumboimbela [60]. 

### 4.5. Flavonoids in Skin Cancer Treatment

*Skin cancer* is one of the most dangerous diseases worldwide, and its incidence is still increasing. Skin cancers include Non-melanoma Skin Cancer (NMSC) and Cutaneous Malignant Melanoma (CMM). The most prevalent skin cancers are NMSC, 90% of which are basal cell carcinoma (BCC) and squamous cell carcinoma (SCC). In addition to BCC and SCC, non-melanoma skin cancer includes cutaneous lymphomas, Merkel cell carcinomas (MCC), and adnexal tumors [123]. BCC, the least aggressive type of NMSC, forms in basal cells in the middle epidermis layer. Despite its characteristics, such as the ability of local spread to other parts, tissue damage, recurrence, and restricted metastatic potential, BCC shows a low degree of malignancy. On the other hand, SCC is characterized by an abnormal proliferation of invasive squamous cells that can metastasize. SCCs exhibit significant potential for recurrence, depending on the size of the tumor, the depth of the lesion, the degree of histological differentiation, as well as on the anatomical location [124]. Early detection of BCC and SCC is important in treating these kinds of cancer with an excellent prognosis. Instead of surgical excision, which is still the first-choice therapy in NMSC treatment, there are many other approaches, such as cryotherapy, photodynamic therapy, topical diclofenac sodium 3%, and topical imiquimod 5% [125]. Cutaneous Malignant Melanoma (CMM), with a relatively low incidence, is considered the most aggressive type of skin cancer. CMM has the highest mortality of all skin cancers. When considering people with skin cancer, CMM is responsible for approximately 80% of deaths because of its high potential to invade and metastasize [123,126,127]. The risk factors for CMM include exposure to ultraviolet radiation, fair skin, fair hair, and a family history of the disease, including genetic susceptibility [128]. Early stages of melanoma could be treated by surgery, but based on the stage, treatment includes immunotherapy, chemotherapy, radiation therapy, targeted therapy, and also their combinations [129]. The main problem in melanoma treatment is its heterogeneity, resistance to therapy, and side effects of used drugs. Therefore, it is important to develop more efficient anticancer therapy. Recently, there has been growing evidence about the positive effect of phytochemicals, including flavonoids, used in cancer treatment in combination with used therapies, where phytochemicals could reduce adverse effects, such as anticancer and antimutagenic compounds altering cellular pathways that are associated with cell growth, cell proliferation, influence the cell cycle, inhibits angiogenesis, proliferation, and activation of proapoptotic proteins [130,131,132].

**Figure 3:**
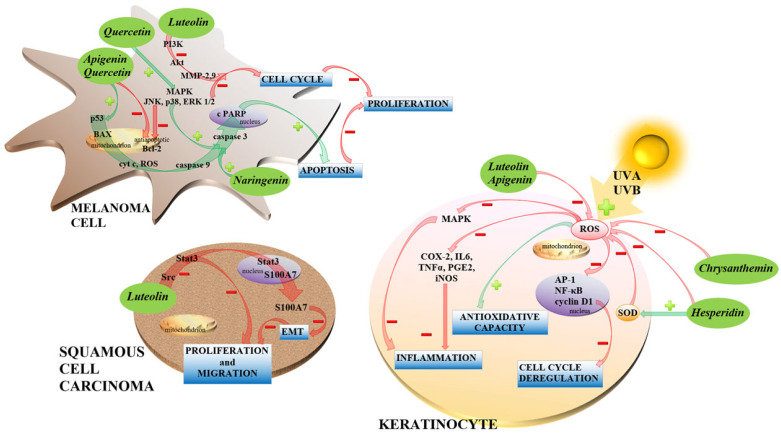
Possible mechanisms of action of flavonoids in melanoma cells, squamous cell carcinoma, and UV-protection of keratinocytes. Anticancer effects are primarily based on activating proapoptotic and antiproliferative signaling cascades. The photoprotective mechanism consists in reducing UV-induced reactive oxygen species production and the subsequent increase in antioxidative capacity, anti-inflammatory effect and inhibition of the deregulation of essential cellular functions.

In addition to the possible use of flavonoids in cancer treatment, there is much evidence for many positive effects against various types of damage, which can represent a stimulus for carcinogenesis. Thus, the effects of the flavonoids can be cancer-cytotoxic, cancer treatment-promoting, or cancer-preventing (Figure 3).

***Naringenin*** has been studied as a possible compound in melanoma cancer treatment. Choi et al. [53] investigated the anticancer effect against B16F10 murine and SK-MEL-28 human melanoma cell lines. It was demonstrated that naringenin induced tumor cell death via activation of the cells’ caspase 3/PARP apoptotic pathway. Moreover, naringenin inhibits melanoma cancer cell proliferation and migration in a dose-dependent manner, supported by inhibiting ERK1/2 and JNK MAPK protein phosphorylation [53].

***Luteolin*** (3,4,5,7-tetrahydroxy flavone) is another promising molecule in skin cancer research tested on animal models and various cell line types. The preventive effect of luteolin was demonstrated in the JB6P+ cell line, in which luteolin suppressed UVB-induced COX-2 (cyclooxygenase-2) expression, AP-1 (activator protein-1), and NF-κB activity. Moreover, luteolin binds directly to protein kinase C epsilon type (PKCε) and Src in an ATP-competitive manner, thus inhibiting UVB-induced MAPK and Akt signaling pathway phosphorylation. In the SKH-1 hairless mouse model, luteolin showed similar molecular effects leading to tumor incidence, multiplicity, and size suppression [38]. The anticancer mechanism of luteolin was also studied in A375 human melanoma cells. It was shown that luteolin significantly inhibits the proliferation, migration, and invasion of A375 cells and induces cell death in a concentration-dependent manner. Luteolin decreased the expression of matrix metallopeptidase 2 (MMP-2) and matrix metallopeptidase 9 (MMP-9) by the phosphatidyl inositol 3-kinase/protein kinase B (PI3K/Akt) pathway [39]. Protein S100A7 is an important activator of epithelial–mesenchymal transition, which significantly impacts tumorigenesis and metastatic potential of cancer cells. In squamous carcinoma cell line A431-III, luteolin is effective in decreasing the levels of S100A7 by inhibiting Src/Stat3 signaling [40].

***Apigenin*** (4′,5,7-trihydroxyflavone) can be considered a potential carcinostatic agent. Woo et al. [36] studied the effect of apigenin on cell proliferation and apoptosis of A375P and A375SM human melanoma cell lines. It was observed that both the migration ability decrease and the apoptotic rate increase were dose-dependent in both melanoma cell lines. The cell growth was inhibited as a result of the activation of apoptotic pathways related to increased levels of apoptotic proteins p53, BAX, cleaved caspase 9, and cleaved PARP and a decreased level of anti-apoptotic protein Bcl-2. According to the MAPK (ERK, JNK, and p38) and Akt signaling pathways, the proteins were differentially expressed in the two melanoma cell lines in response to apigenin treatment, and dose dependence was not confirmed. In the A375SM cell line-derived xenograft mouse model, authors proved the reduction in tumor size after apigenin treatment [36]. According to the results of several studies, apigenin may exhibit both pro- and anti-oxidant effects on various cancer types. While in skin cancerogenesis, it shows a protective effect via a decrease in ROS, which modulates the activation of MAPK and AP-1 in keratinocytes and finally leads to the repression of MMP-1-promoting skin cancer [37], in certain cancer cell types, it also exerts prooxidative effects leading to glutathione depletion and superoxide dismutase inhibition [41].

***Quercetin***, as a bioactive compound, shows promising potential in preventing and treating melanoma. The anticancer activity of quercetin was studied against a B16 murine melanoma cell line. Quercetin inhibited cell proliferation, induced apoptosis, and reduced the proportion of cells in the S and G2/M phases of the cell cycle [48]. Kim et al. [49] evaluated the anticancer effect of quercetin on A375SM and A375P human melanoma cells. It was observed that quercetin induced apoptosis via increased expression of proapoptotic BAX, cleaved PARP, and decreased expression of anti-apoptotic protein Bcl-2. Expression of proteins related to proliferation and differentiation 14hosphor-JNK, 13hosphor-p38, and 14hosphor-ERK1/2 were dose-dependent after quercetin treatment. Their study observed a reduction in tumor volume in the A375SM cell line-derived xenograft mouse model after quercetin treatment [49].

There is cumulating evidence about the effect of flavonoids against UV light damage. Besides the already mentioned flavon luteolin, compounds from flavanol and anthocyanidin groups may provide UV protection. The flavanol ***hesperidin*** (7-*O*-glycoside of hesperetin-(2*S*)-3′,5,7-trihydroxy-4′-methoxyflavan-4-one) can be found in citrus fruits, exhibits a direct protective effect on the UVA irradiation-induced damage in human keratinocytes via reduction of oxidative stress and inflammatory response. Hesperidin treatment increases SOD levels and total antioxidative capacity levels and decreases malondialdehyde levels in both control and UVA-irradiated HaCaT cells. Moreover, hesperidin treatment decreases proinflammatory cytokines IL-6, IL-1β, and TNF-α on both mRNA and protein expression levels in UVA-irradiated cells [55].

Another protagonist in UV protection is blue anthocyanidin ***chrysanthemin*** (3-*O*-glucoside of cyanidin-2-(3,4-dihydroxyphenyl)chromenylium-3,5,7-triol) present in blackcurrant, raspberries, peaches, or red oranges. Chrysanthemin inhibits UVB irradiation-induced damage and inflammation in animal experiments via inhibiting glutathione depletion, lipid peroxidation, and myeloperoxidation. Among other positive effects were the decreased production of proinflammatory cytokines IL6 and TNF-α, reduced phosphorylation of various MAP kinases involved in inflammatory response (Erk1/2, p38, JNK1/2, MKK4), other inflammation-related molecules COX-2, PGE2, iNOS, but also inhibition of cyclin D1, nuclear translocation of transcription factor NF-κB, and the decrease in markers of DNA-oxidative stress (PCNA—proliferating cell nuclear antigen, 8-hydroxy-2′-deoxyguanosine). The protection provided by chrysanthemin against inflammation and cancer thus can be substantial and manifested on several levels [58].

Additionally, using ***flavonoid extracts*** of various origins could represent an exciting application. Extracts have the potential to be more efficient due to the synergistic action of active compounds. Promising results are mainly in the field of prevention of cancerogenesis. The protective effect of ***grape seed proanthocyanidins*** (GSPs) on epidermal keratinocytes has been described for a long time. GSPs are a mixture of several polyphenols/flavanols and mainly contain proanthocyanidins (89%), which constitute dimers, trimers, tetramers, oligomers, and monomeric flavanols (6.6%). GSPs’ treatment inhibits UVB-induced lipid peroxidation, protein oxidation, and DNA damage, scavenges hydroxyl radicals and superoxide anions, and inhibits the depletion of the antioxidant defense components (glutathione peroxidase, catalase, superoxide dismutase, and glutathione). Based on experiments, the proposed mechanism is the inhibition of UVB-induced MAPK (ERK 1/2, JNK, p38) phosphorylation, which is caused mainly by UV-induced H_2_O_2_. Moreover, GSPs also inhibit UVB-induced activation of IκBα and IKKα, which are mediators of activation of the NF-κB/p pathway [133]. The *Myrica rubra* fruit extracts with the content of effective compounds ***myricetin-O-deoxyhexoside, quercetin-O deoxyhexoside,*** and ***kaempferol-o-hexoside*** have no apparent cytotoxic effect on melanoma cell lines (B16-F0 and A2058), but water extracts may inhibit ROS production. Moreover, it can also inhibit melanogenesis via direct reversible inhibition of tyrosine kinase activity and down-regulation of melanogenesis-related genes MITF and TRP-1 leading to a decrease in the expression of the corresponding proteins [134]. Herbs widely used as tea beverages with high flavonoid content with possible effects on skin cancers are interesting. Among such belong the green tea (*Camellia sinensis*) leaves’ rich in flavanols (catechin derivatives), rooibos’ (*Aspalathus linearis*) rich in flavanols, and dihydrochalcones’ (***aspalathin*** and ***nothofagin***), or honeybushes’ (*Cyclopia* sp.—*C. intermedia* and *C. subternata*) rich in flavanols and flavanones (***hesperidin***). Rooibos extracts (water and methanol) and the aqueous extracts of honeybush alter cellular growth by a similar mechanism to green tea extracts, which involves the disruption of metabolic activity in the cell via mitochondrial membrane depolarization. Cell proliferation is inhibited at a lower concentration, while apoptosis is induced at a higher concentration, primarily targeting precancerous cells. The chemopreventive properties of herbal teas are predominant due to specific polyphenol-cell interactions and are extract-unique, reflecting the diversity of polyphenols in plants [135].

## 5. Conclusions

Human skin is considered the largest organ with the role of covering and protecting the body. The skin has many irreplaceable functions; for instance, it is the body’s first line of protection against external factors and pathogens, regulating the temperature, amount of water, and many others. As the largest organ, the skin is affected by many diseases, some minor or rare, but some are very common with typical symptoms. Skin diseases negatively impact life quality, so their targeted treatment without or with limited side effects is very important. Recently, there has been growing evidence on using natural substances, including flavonoids, in treating various skin diseases. Flavonoids are widely used in in vitro and in vivo studies and in clinical trials with significant positive results in treating skin diseases. Most of the studies were performed in vitro or in animal experiments; thus, more in vivo human experiments are necessary. For progress in this field, it is important to continue research, determine the appropriate dosage, evaluate the safety of the phytochemicals used, and rule out their possible side effects.

## Figures and Tables

**Table 1:** Plant sources of flavonoids.

Flavonoid Group	Representatives	Plant Source [26,27,28]	Application in Skin Diseases Treatment
Flavones, isoflavones	apigenin, luteolin, chrysin, genistein, baicalein, neobavaisoflavone, licoflavone A, nobiletin	parsley, oregano, licorice, *Cleome* sp.	vitiligo [29] psoriasis [30,31,32,33] acne vulgaris [34,35], skin cancer [36,37,38,39,40,41]
Flavonols	quercetin, kaempferol, rhamnetin, and fisetin, rutin, myricetin, kaempferide	capers, parsley, elderberry, sorrel, *Embelia ribes Burm*	vitiligo [29,42] psoriasis [43] acne vulgaris [44,45,46], atopic dermatitis [47] skin cancer [48,49]
Flavanones	naringin, naringenin, liquiritigenin	oregano, licorice	atopic dermatitis [50,51,52] skin cancer [53]
Flavanonols	astilbin	*Smilax* sp., *Rhododendron simsii*, *Saccharomyces cerevisiae*	psoriasis [54]
Flavanols or catechins	epigallocatechin, hesperidin	green tea, black tea, oolong, white tea, dark chocolate, cranberries, strawberries, blackberries, kiwis, cherries, pears, peaches, apples, avocados	skin cancer [55]
Anthocyanidins	*delphinidin, chrysanthemin*	elderberry, aronia, bilberries, chickpeas, blackcurrant, eggplant, roselle, grape	psoriasis [56,57] skin cancer [58]
Neoflavonoids	*calophyllolide*, *dalbergichromene*, *dalbergin*, *nivetin*, *coutareagenin*	*Calophyllum inophyllum* (tamanu oil), *Mesua thwaitesii*, *Dalbergia sissoo*, *Hintonia latiflora*, *Echinops niveus*	atopic dermatitis [59,60]
Chalcones	*licochalcone A*, *licochalcone C*, *isoliquiritigenin*	licorice	acne vulgaris [34]

## Data Availability

Not applicable.

## References

[1] Webb D. (2013). Phytochemicals’ Role in Good Health. Today’s Dietit..

[2] Liu R.H. (2013). Dietary bioactive compounds and their health implications. J. Food Sci..

[3] Cao H., Chai T.T., Wang X., Morais-Braga M.F.B., Yang J.H., Wong F.C., Wang R., Yao H., Cao J., Cornara L. (2017). Phytochemicals from fern species: Potential for medicine applications. Phytochem. Rev..

[4] Yu M., Gouvinhas I., Rocha J., Barros A.I.R.N.A. (2021). Phytochemical and antioxidant analysis of medicinal and food plants towards bioactive food and pharmaceutical resources. Sci. Rep..

[5] Nollet L.M., Gutierrez-Uribe J.A. (2018). Phenolic Compounds in Food: Characterization and Analysis.

[6] Singh D., Chaudhuri P.K. (2018). A review on phytochemical and pharmacological properties of Holy basil (*Ocimum sanctum* L.). Ind. Crops Prod..

[7] Liu R.H. (2004). Potential synergy of phytochemicals in cancer prevention: Mechanism of action. J. Nutr..

[8] Giordano E., Dávalos A., Visioli F. (2014). Chronic hydroxytyrosol feeding modulates glutathione-mediated oxido-reduction pathways in adipose tissue: A nutrigenomic study. Nutr. Metab. Cardiovasc. Dis..

[9] Nicod N., Chiva-Blanch G., Giordano E., Dávalos A., Parker R.S., Visioli F. (2014). Green tea, cocoa, and red wine polyphenols moderately modulate intestinal inflammation and do not increase high-density lipoprotein (HDL) production. J. Agric. Food Chem..

[10] Ain H.B.U., Saeed F., Barrow C.J., Dunshea F.R., Suleria H.A.R., Murthy H.N., Bapat V.A. (2020). Food processing waste: A potential source for bioactive compounds. Bioactive Compounds in Underutilized Fruits and Nuts.

[11] Brahem M., Renard C.M., Eder S., Loonis M., Ouni R., Mars M., Le Bourvellec C. (2017). Characterization and quantification of fruit phenolic compounds of european and tunisian pear cultivars. Food Res. Int..

[12] Suleria H.A.R., Barrow C.J., Dunshea F.R. (2020). Screening and Characterization of Phenolic Compounds and Their Antioxidant Capacity in Different Fruit Peels. Foods.

[13] Ferlemi A.V., Lamari F.N. (2016). Berry Leaves: An Alternative Source of Bioactive Natural Products of Nutritional and Medicinal Value. Antioxidants.

[14] Sahu P.K., Cervera-Mata A., Chakradhari S., Singh Patel K., Towett E.K., Quesada-Granados J.J., Martín-Ramos P., Rufián-Henares J.A. (2022). Seeds as Potential Sources of Phenolic Compounds and Minerals for the Indian Population. Molecules.

[15] Miller F.W. (2023). The increasing prevalence of autoimmunity and autoimmune diseases: An urgent call to action for improved understanding, diagnosis, treatment, and prevention. Curr. Opin. Immunol..

[16] Chang R., Yen-Ting Chen T., Wang S.I., Hung Y.M., Chen H.Y., Wei C.J. (2023). Risk of autoimmune diseases in patients with COVID-19: A retrospective cohort study. EClinicalMedicine.

[17] Mutha R.E., Tatiya A.U., Surana S.J. (2021). Flavonoids as natural phenolic compounds and their role in therapeutics: An overview. Futur J. Pharm Sci..

[18] Karak P. (2019). Biological activities of flavonoids: An overview. Int. J. Pharm. Sci. Res..

[19] Ma E.Z., Khachemoune A. (2022). Flavonoids and their therapeutic applications in skin diseases. Arch. Dermatol. Res..

[20] Liu K., Luo M., Wei S. (2019). The Bioprotective Effects of Polyphenols on Metabolic Syndrome against Oxidative Stress: Evidences and Perspectives. Oxid. Med. Cell. Longev..

[21] Gębka N., Adamczyk J., Gębka-Kępińska B., Mizgała-Izworska E. (2022). The role of flavonoids in prevention and treatment of selected skin diseases. J. Pre-Clin. Clin. Res..

[22] Pietta P. (2000). Flavonoids as antioxidants. J. Nat. Prod..

[23] Kumar S., Pandey A.K. (2013). Chemistry and biological activities of flavonoids: An overview. Sci. World J..

[24] Ahmed S.I., Hayat M.Q., Tahir M., Mansoor Q., Ismail M., Keck K., Bates R.B. (2016). Pharmacologically active flavonoids from the anticancer, antioxidant and antimicrobial extracts of *Cassia angustifolia* Vahl. BMC Complement. Altern. Med..

[25] Panche A.N., Diwan A.D., Chandra S.R. (2016). Flavonoids: An overview. J. Nutr. Sci..

[26] Waheed Janabi A.H., Kamboh A.A., Saeed M., Xiaoyu L., BiBi J., Majeed F., Naveed M., Mugha M.J., Korejo N.A., Kamboh R. (2020). Flavonoid-rich foods (FRF): A promising nutraceutical approach against lifespan-shortening diseases. Iran J. Basic Med. Sci..

[27] Sharma V., Gautam D.N.S., Radu A.F., Behl T., Bungau S.G., Vesa C.M. (2022). Reviewing the Traditional/Modern Uses, Phytochemistry, Essential Oils/Extracts and Pharmacology of *Embelia ribes* Burm. Antioxidants.

[28] Khuntia A., Martorell M., Ilango K., Bungau S.G., Radu A.F., Behl T., Sharifi-Rad J. (2022). Theoretical evaluation of Cleome species’ bioactive compounds and therapeutic potential: A literature review. Biomed. Pharmacother..

[29] Takekoshi S., Nagata H., Kitatani K. (2014). Flavonoids enhance melanogenesis in human melanoma cells. Tokai J. Exp. Clin. Med..

[30] Weng Z., Patel A.B., Vasiadi M., Therianou A., Theoharides T.C. (2014). Luteolin inhibits human keratinocyte activation and decreases NF-κB induction that is increased in psoriatic skin. PLoS ONE.

[31] Zhou W., Hu M., Zang X., Liu Q., Du J., Hu J., Zhang L., Du Z., Xiang Z. (2020). Luteolin attenuates imiquimod-induced psoriasis-like skin lesions in BALB/c mice via suppression of inflammation response. Biomed. Pharmacother..

[32] Hung C.H., Wang C.N., Cheng H.H., Liao J.W., Chen Y.T., Chao Y.W., Jiang J.L., Lee C.C. (2018). Baicalin Ameliorates Imiquimod-Induced Psoriasis-Like Inflammation in Mice. Planta Med..

[33] Wang P.W., Lin T.Y., Yang P.M., Fang J.Y., Li W.T., Pan T.L. (2022). Therapeutic efficacy of Scutellaria baicalensis Georgi against psoriasis-like lesions via regulating the responses of keratinocyte and macrophage. Biomed. Pharmacother..

[34] Ruan S.F., Hu Y., Wu W.F., Du Q.Q., Wang Z.X., Chen T.T., Shen Q., Liu L., Jiang C.P., Li H. (2022). Explore the Anti-Acne Mechanism of Licorice Flavonoids Based on Metabonomics and Microbiome. Front. Pharmacol..

[35] Sato T., Takahashi A., Kojima M., Akimoto N., Yano M., Ito A. (2007). A citrus polymethoxy flavonoid, nobiletin inhibits sebum production and sebocyte proliferation, and augments sebum excretion in hamsters. J. Investig. Dermatol..

[36] Woo J.S., Choo G.S., Yoo E.S., Kim S.H., Lee J.H., Han S.H., Kim H.J., Jung S.H., Park Y.S., Kim B.S. (2020). Apigenin induces apoptosis by regulating Akt and MAPK pathways in human melanoma cell A375SM. Mol. Med. Rep..

[37] Hwang Y.P., Oh K.N., Yun H.J., Jeong H.G. (2011). The flavonoids apigenin and luteolin suppress ultraviolet A-induced matrix metalloproteinase-1 expression via MAPKs and AP-1-dependent signaling in HaCaT cells. J. Dermatol. Sci..

[38] Byun S., Lee K.W., Jung S.K., Lee E.J., Hwang M.K., Lim S.H., Bode A.M., Lee H.J., Dong Z. (2010). Luteolin inhibits protein kinase C(epsilon) and c-Src activities and UVB-induced skin cancer. Cancer Res..

[39] Yao X., Jiang W., Yu D., Yan Z. (2019). Luteolin inhibits proliferation and induces apoptosis of human melanoma cells in vivo and in vitro by suppressing MMP-2 and MMP-9 through the PI3K/AKT pathway. Food Funct..

[40] Fan J.J., Hsu W.H., Lee K.H., Chen K.C., Lin C.W., Lee Y.A., Ko T.P., Lee L.T., Lee M.T., Chang M.S. (2019). Dietary Flavonoids Luteolin and Quercetin Inhibit Migration and Invasion of Squamous Carcinoma through Reduction of Src/Stat3/S100A7 Signaling. Antioxidants.

[41] Slika H., Mansour H., Wehbe N., Nasser S.A., Iratni R., Nasrallah G., Shaito A., Ghaddar T., Kobeissy F., Eid A.H. (2022). Therapeutic potential of flavonoids in cancer: ROS-mediated mechanisms. Biomed. Pharmacother..

[42] Li J., Yang M., Song Y. (2022). Molecular mechanism of vitiligo treatment by bailing tablet based on network pharmacology and molecular docking. Medicine.

[43] Chen H., Lu C., Liu H., Wang M., Zhao H., Yan Y., Han L. (2017). Quercetin ameliorates imiquimod-induced psoriasis-like skin inflammation in mice via the NF-κB pathway. Int. Immunopharmacol..

[44] Lim Y.H., Kim I.H., Seo J.J. (2007). In vitro activity of kaempferol isolated from the Impatiens balsamina alone and in combination with erythromycin or clindamycin against Propionibacterium acnes. J. Microbiol..

[45] Lim H.J., Kang S.H., Song Y.J., Jeon Y.D., Jin J.S. (2021). Inhibitory Effect of Quercetin on Propionibacterium acnes-induced Skin Inflammation. Int. Immunopharmacol..

[46] Amer S.S., Nasr M., Abdel-Aziz R.T.A., Moftah N.H., El Shaer A., Polycarpou E., Mamdouh W., Sammour O. (2020). Cosm-nutraceutical nanovesicles for acne treatment: Physicochemical characterization and exploratory clinical experimentation. Int. J. Pharm..

[47] Hou D.D., Zhang W., Gao Y.L., Sun Y.Z., Wang H.X., Qi R.Q., Chen H.D., Gao X.H. (2019). Anti-inflammatory effects of quercetin in a mouse model of MC903-induced atopic dermatitis. Int. Immunopharmacol..

[48] Soll F., Ternent C., Berry I.M., Kumari D., Moore T.C. (2020). Quercetin Inhibits Proliferation and Induces Apoptosis of B16 Melanoma Cells In Vitro. Assay Drug Dev. Technol..

[49] Kim S.H., Yoo E.S., Woo J.S., Han S.H., Lee J.H., Jung S.H., Kim H.J., Jung J.Y. (2019). Antitumor and apoptotic effects of quercetin on human melanoma cells involving JNK/P38 MAPK signaling activation. Eur. J. Pharmacol..

[50] Kim T.H., Kim G.D., Ahn H.J., Cho J.J., Park Y.S., Park C.S. (2013). The inhibitory effect of naringenin on atopic dermatitis induced by DNFB in NC/Nga mice. Life Sci..

[51] Lee H.S., Kim E.N., Jeong G.S. (2020). Oral Administration of Liquiritigenin Confers Protection from Atopic Dermatitis through the Inhibition of T Cell Activation. Biomolecules.

[52] Karuppagounder V., Arumugam S., Thandavarayan R.A., Sreedhar R., Giridharan V.V., Pitchaimani V., Afrin R., Harima M., Krishnamurthy P., Suzuki K. (2016). Naringenin ameliorates skin inflammation and accelerates phenotypic reprogramming from M1 to M2 macrophage polarization in atopic dermatitis NC/Nga mouse model. Exp. Dermatol..

[53] Choi J., Lee D.H., Jang H., Park S.Y., Seol J.W. (2020). Naringenin exerts anticancer effects by inducing tumor cell death and inhibiting angiogenesis in malignant melanoma. Int. J. Med. Sci..

[54] Xu Q., Liu Z., Cao Z., Shi Y., Yang N., Cao G., Zhang C., Sun R., Zhang C. (2022). Topical astilbin ameliorates imiquimod-induced psoriasis-like skin lesions in SKH-1 mice via suppression dendritic cell-Th17 inflammation axis. J. Cell. Mol. Med..

[55] Li M., Lin X.F., Lu J., Zhou B.R., Luo D. (2016). Hesperidin ameliorates UV radiation-induced skin damage by abrogation of oxidative stress and inflammatory in HaCaT cells. J. Photochem. Photobiol. B.

[56] Pal H.C., Chamcheu J.C., Adhami V.M., Wood G.S., Elmets C.A., Mukhtar H., Afaq F. (2015). Topical application of delphinidin reduces psoriasiform lesions in the flaky skin mouse model by inducing epidermal differentiation and inhibiting inflammation. Br. J. Dermatol..

[57] Chamcheu J.C., Pal H.C., Siddiqui I.A., Adhami V.M., Ayehunie S., Boylan B.T., Noubissi F.K., Khan N., Syed D.N., Elmets C.A. (2015). Prodifferentiation, anti-inflammatory and antiproliferative effects of delphinidin, a dietary anthocyanidin, in a full-thickness three-dimensional reconstituted human skin model of psoriasis. Skin Pharmacol. Physiol..

[58] Pratheeshkumar P., Son Y.O., Wang X., Divya S.P., Joseph B., Hitron J.A., Wang L., Kim D., Yin Y., Roy R.V. (2014). Cyanidin-3-glucoside inhibits UVB-induced oxidative damage and inflammation by regulating MAP kinase and NF-κB signaling pathways in SKH-1 hairless mice skin. Toxicol. Appl. Pharmacol..

[59] Pribowo A., Girish J., Gustiananda M., Nandhira R.G., Hartrianti P. (2021). Potential of Tamanu (Calophyllum inophyllum) Oil for Atopic Dermatitis Treatment. Evid.-Based Complement Altern. Med..

[60] Tumboimbela J.R.W. (2023). The Study of Tamanu (Calophyllum inophyllum) Oil Effects on Atopic Dermatitis-Related Gene Expression Levels on TNF-α and IFN-γ Induced HaCaT Cells. Bachelor’s Thesis.

[61] Ferreira M.S., Magalhães M.C., Oliveira R., Sousa-Lobo J.M., Almeida I.F. (2021). Trends in the Use of Botanicals in Anti-Aging Cosmetics. Molecules.

[62] De Oliveira C.A., Dario M.F., Martínez L., Kharissova O., Kharisov B. (2019). Bioactive Cosmetics. Handbook of Ecomaterials.

[63] Antignac E., Nohynek G.J., Re T., Clouzeau J., Toutain H. (2011). Safety of botanical ingredients in personal care products/cosmetics. Food Chem. Toxicol..

[64] Othmer K. (2012). Chemical Technology of Cosmetics.

[65] Zillich O.V., Schweiggert-Weisz U., Eisner P., Kerscher M. (2015). Polyphenols as active ingredients for cosmetic products. Int. J. Cosmet. Sci..

[66] Hoang H.T., Moon J.-Y., Lee Y.-C. (2021). Natural Antioxidants from Plant Extracts in Skincare Cosmetics: Recent Applications, Challenges and Perspectives. Cosmetics.

[67] Arct J., Pytkowska K. (2008). Flavonoids as components of biologically active cosmeceuticals. Clin. Dermatol..

[68] Hajheidari Z., Saeedi M., Morteza-Semnani K., Soltani A. (2013). Effect of *Aloe vera* topical gel combined with tretinoin in treatment of mild and moderate acne vulgaris: A randomized, double-blind, prospective trial. J. Dermatol. Treat..

[69] Yousef H., Alhajj M., Sharma S. (2022). Anatomy, Skin (Integument), Epidermis.

[70] Dyring-Andersen B., Løvendorf M.B., Coscia F., Santos A., Møller L.B.P., Colaço A.R., Niu L., Bzorek M., Doll S., Andersen J.L. (2020). Spatially and cell-type resolved quantitative proteomic atlas of healthy human skin. Nat. Commun..

[71] Goldsmith L.A. (1990). My organ is bigger than your organ. Arch. Dermatol..

[72] Herman A., Herman A.P. (2019). Antimicrobial peptides activity in the skin. Skin Res. Technol..

[73] Tobin D.J. (2006). Biochemistry of human skin—Our brain on the outside. Chem. Soc. Rev..

[74] Elpa D.P., Chiu H.Y., Wu S.P., Urban P.L. (2021). Skin Metabolomics. Trends Endocrinol. Metab..

[75] Griffin M., Casadio R., Bergamini C.M. (2002). Transglutaminases: Nature’s biological glues. Biochem. J..

[76] Chuong C.M., Nickoloff B.J., Elias P.M., Goldsmith L.A., Macher E., Maderson P.A., Sundberg J.P., Tagami H., Plonka P.M., Thestrup-Pederson K. (2002). What is the ‘true’ function of skin?. Exp. Dermatol..

[77] Coderch L., Lopez O., de la Maza A., Parra J.L. (2003). Ceramides and skin function. Am J. Clin. Dermatol..

[78] Brucker-Tuderman L., Bolognia J.L., Jorizzo J.L., Rapini R.P. (2003). Biology of the Extracellular Matrix, Dermatology.

[79] Tracy L.E., Minasian R.A. (2016). Caterson EJ. Extracellular Matrix and Dermal Fibroblast Function in the Healing Wound. Adv. Wound Care.

[80] Uitto J. (1989). Connective tissue biochemistry of the aging dermis: Age-associated alterations in collagen and elastin. Clin. Geriatr. Med..

[81] Freinkel R.K., Woodley D.T. (2001). The Biology of Skin.

[82] Chen J., Liu Y., Zhao Z., Qiu J. (2021). Oxidative stress in the skin: Impact and related protection. Int. J. Cosmet. Sci..

[83] Xian D., Guo M., Xu J., Yang Y., Zhao Y., Zhong J. (2021). Current evidence to support the therapeutic potential of flavonoids in oxidative stress-related dermatoses. Redox Rep..

[84] Rubio C.P., Cerón J.J. (2021). Spectrophotometric assays for evaluation of Reactive Oxygen Species (ROS) in serum: General concepts and applications in dogs and humans. BMC Vet. Res..

[85] Kruk J., Duchnik E. (2014). Oxidative stress and skin diseases: Possible role of physical activity. Asian Pac. J. Cancer Prev..

[86] Tsuchida K., Kobayashi M. (2020). Oxidative stress in human facial skin observed by ultraweak photon emission imaging and its correlation with biophysical properties of skin. Sci. Rep..

[87] Britannica, The Editors of Encyclopaedia (2022). “Ultraviolet Radiation”. Encyclopedia Britannica. https://www.britannica.com/science/ultraviolet-radiation.

[88] Zastrow L., Groth N., Klein F., Kockott D., Lademann J., Renneberg R., Ferrero L. (2009). The missing link–light-induced (280–1600 nm) free radical formation in human skin. Skin Pharmacol. Physiol..

[89] Marrot L. (2018). Pollution and Sun Exposure: A Deleterious Synergy. Mechanisms and Opportunities for Skin Protection. Curr. Med. Chem..

[90] Stefanovic N., Flohr C., Irvine A.D. (2020). The exposome in atopic dermatitis. Allergy.

[91] Patra V., Byrne S.N., Wolf P. (2016). The Skin Microbiome: Is It Affected by UV-induced Immune Suppression?. Front. Microbiol..

[92] Seneschal J., Boniface K., D’Arino A., Picardo M. (2021). An Update on Vitiligo Pathogenesis. Pigment. Cell Melanoma Res..

[93] Pang Y., Wu S., He Y., Nian Q., Lei J., Yao Y., Guo J., Zeng J. (2021). Plant Derived Compounds as Promising Therapeutics for Vitiligo. Front. Pharmacol..

[94] Shivasaraun U.V., Sureshkumar R., Karthika C., Puttappa N. (2018). Flavonoids as adjuvant in psoralen based photochemotherapy in the management of vitiligo/leucoderma. Med. Hypotheses.

[95] Gianfaldoni S., Tchernev G., Lotti J., Wollina U., Satolli F., Rovesti M., França K., Lotti T. (2018). Unconventional Treatments for Vitiligo: Are They (Un) Satisfactory?. Open Access Maced. J. Med. Sci..

[96] Nagata H., Takekoshi S., Takeyama R., Homma T., Yoshiyuki Osamura R. (2004). Quercetin enhances melanogenesis by increasing the activity and synthesis of tyrosinase in human melanoma cells and in normal human melanocytes. Pigment Cell Res..

[97] Wang J.Y., Chen H., Wang Y.Y., Wang X.Q., Chen H.Y., Zhang M., Tang Y., Zhang B. (2017). Network pharmacological mechanisms of *Vernonia anthelmintica* (L.) in the treatment of vitiligo: Isorhamnetin induction of melanogenesis via up-regulation of melanin-biosynthetic genes. BMC Syst. Biol..

[98] Cannavò S.P., Riso G., Casciaro M., Di Salvo E., Gangemi S. (2019). Oxidative stress involvement in psoriasis: A systematic review. Free Radic. Res..

[99] Nair P.A., Badri T. (2022). Psoriasis.

[100] World Health Orgnisation (2016). Global Report on Psoriasis. https://apps.who.int/iris/bitstream/handle/10665/204417/9789241565189_eng.pdf.psoriasis?sequence=1.

[101] Guarneri F., Bertino L., Pioggia G., Casciaro M., Gangemi S. (2021). Therapies with Antioxidant Potential in Psoriasis, Vitiligo, and Lichen Planus. Antioxidants.

[102] Nowak-Perlak M., Szpadel K., Jabłόnska I., Pizon M., Wόzniak M. (2022). Promising Strategies in Plant-Derived Treatments of Psoriasis-Update of In Vitro, In Vivo, and Clinical Trials Studies. Molecules.

[103] Gendrisch F., Esser P.R., Schempp C.M., Wölfle U. (2021). Luteolin as a modulator of skin aging and inflammation. Biofactors.

[104] Tungmunnithum D., Thongboonyou A., Pholboon A., Yangsabai A. (2018). Flavonoids and Other Phenolic Compounds from Medicinal Plants for Pharmaceutical and Medical Aspects: An Overview. Medicines.

[105] Tan A.U., Schlosser B.J., Paller A.S. (2017). A review of diagnosis and treatment of acne in adult female patients. Int. J. Womens Dermatol..

[106] Bhate K., Williams H.C. (2013). Epidemiology of acne vulgaris. Br. J. Dermatol..

[107] Williams H.C., Dellavalle R.P., Garner S. (2012). Acne vulgaris. Lancet.

[108] Falcocchio S., Ruiz C., Pastor F.I.J., Saso L., Diaz P. (2006). Propionibacterium acnes GehA lipase, an enzyme involved in acne development, can be successfully inhibited by defined natural substances. J. Mol. Catal. B Enzym..

[109] Lu P.H., Hsu C.H. (2016). Does supplementation with green tea extract improve acne in post-adolescent women? A randomized, double-blind, and placebo-controlled clinical trial. Complement Ther. Med..

[110] Yoon J.Y., Kwon H.H., Min S.U., Thiboutot D.M., Suh D.H. (2013). Epigallocatechin-3-gallate improves acne in humans by modulating intracellular molecular targets and inhibiting P. acnes. J. Investig. Dermatol..

[111] Avena-Woods C. (2017). Overview of atopic dermatitis. Am. J. Manag. Care.

[112] Nutten S. (2015). Atopic Dermatitis: Global Epidemiology and Risk Factors. Ann. Nutr. Metab..

[113] Hadi H.A., Tarmizi A.I., Khalid K.A., Gajdács M., Aslam A., Jamshed S. (2021). The Epidemiology and Global Burden of Atopic Dermatitis: A Narrative Review. Life.

[114] Hussain Z., Thu H.E., Shuid A.N., Kesharwani P., Khan S., Hussain F. (2017). Phytotherapeutic potential of natural herbal medicines for the treatment of mild-to-severe atopic dermatitis: A review of human clinical studies. Biomed. Pharmacother..

[115] Kim J., Kim B.E., Leung D.Y.M. (2019). Pathophysiology of atopic dermatitis: Clinical implications. Allergy Asthma Proc..

[116] Cabanillas B., Brehler A.C., Novak N. (2017). Atopic dermatitis phenotypes and the need for personalized medicine. Curr. Opin. Allergy Clin. Immunol..

[117] Silverberg N.B., Silverberg J.I. (2015). Inside out or outside in: Does atopic dermatitis disrupt barrier function or does disruption of barrier function trigger atopic dermatitis?. Cutis.

[118] Pankonien I., Quaresma M.C., Rodrigues C.S., Amaral M.D. (2022). CFTR, Cell Junctions and the Cytoskeleton. Int. J. Mol. Sci..

[119] Novak N., Bieber T., Leung D.Y. (2003). Immune mechanisms leading to atopic dermatitis. J. Allergy Clin. Immunol..

[120] Wu S., Pang Y., He Y., Zhang X., Peng L., Guo J., Zeng J. (2021). A comprehensive review of natural products against atopic dermatitis: Flavonoids, alkaloids, terpenes, glycosides and other compounds. Biomed. Pharmacother..

[121] Boguniewicz M., Fonacier L., Guttman-Yassky E., Ong P.Y., Silverberg J., Farrar J.R. (2018). Atopic dermatitis yardstick: Practical recommendations for an evolving therapeutic landscape. Ann. Allergy Asthma Immunol..

[122] Choi J.K., Jang Y.H., Lee S., Lee S.R., Choi Y.A., Jin M., Choi J.H., Park J.H., Park P.H., Choi H. (2017). Chrysin attenuates atopic dermatitis by suppressing inflammation of keratinocytes. Food Chem. Toxicol..

[123] Juszczak A.M., Wöelfle U., Končić M.Z., Tomczyk M. (2022). Skin cancer, including related pathways and therapy and the role of luteolin derivatives as potential therapeutics. Med. Res. Rev..

[124] Didona D., Paolino G., Bottoni U., Cantisani C. (2018). Non-Melanoma Skin Cancer Pathogenesis Overview. Biomedicines.

[125] Apalla Z., Nashan D., Weller R.B., Castellsagué X. (2017). Skin Cancer: Epidemiology, Disease Burden, Pathophysiology, Diagnosis, and Therapeutic Approaches. Dermatol. Ther..

[126] Saginala K., Barsouk A., Aluru J.S., Rawla P., Barsouk A. (2021). Epidemiology of Melanoma. Med. Sci..

[127] Olbryt M. (2019). Molecular background of skin melanoma development and progression: Therapeutic implications. Adv. Dermatol. Allergol..

[128] Buja A., Rugge M., De Luca G., Bovo E., Zorzi M., De Toni C., Cozzolino C., Vecchiato A., Del Fiore P., Spina R. (2022). Cutaneous Melanoma in Alpine Population: Incidence Trends and Clinicopathological Profile. Curr. Oncol..

[129] Schvartsman G., Taranto P., Glitza I.C., Agarwala S.S., Atkins M.B., Buzaid A.C. (2019). Management of metastatic cutaneous melanoma: Updates in clinical practice. Ther. Adv. Med. Oncol..

[130] Bellan D.L., Biscaia S.M.P., Rossi G.R., Cristal A.M., Gonçalves J.P., Oliveira C.C., Simas F.F., Sabry D.A., Rocha H.A.O., Franco C.R.C. (2020). Green does not always mean go: A sulfated galactan from Codium isthmocladum green seaweed reduces melanoma metastasis through direct regulation of malignancy features. Carbohydr. Polym..

[131] Souto E.B., Sampaio A.C., Campos J.R., Martins-Gomes C., Aires A., Silva A.M., Attaur R. (2019). Chapter 2—Polyphenols for skin cancer: Chemical properties, structure-related mechanisms of action and new delivery systems. Studies in Natural Products Chemistry.

[132] Imran M., Rauf A., Abu-Izneid T., Nadeem M., Shariati M.A., Khan I.A., Imran A., Orhan I.E., Rizwan M., Atif M. (2019). Luteolin, a flavonoid, as an anticancer agent: A review. Biomed. Pharmacother..

[133] Mantena S.K., Katiyar S.K. (2006). Grape seed proanthocyanidins inhibit UV-radiation-induced oxidative stress and activation of MAPK and NF-kappaB signaling in human epidermal keratinocytes. Free Radic. Biol. Med..

[134] Juang L.J., Gao X.Y., Mai S.T., Lee C.H., Lee M.C., Yao C.L. (2019). Safety assessment, biological effects, and mechanisms of Myrica rubra fruit extract for anti-melanogenesis, anti-oxidation, and free radical scavenging abilities on melanoma cells. J. Cosmet. Dermatol..

[135] Magcwebeba T.U., Swart P., Swanevelder S., Joubert E., Gelderblom W.C. (2016). In Vitro Chemopreventive Properties of Green Tea, Rooibos and Honeybush Extracts in Skin Cells. Molecules.

